# Evaluating the quantity and quality of health economic literature in blinding childhood disorders: a systematic literature review

**DOI:** 10.1007/s40273-023-01311-5

**Published:** 2023-11-16

**Authors:** Lucinda J. Teoh, Salomey Kellett, Dipesh E. Patel, Mario Cortina-Borja, Ameenat Lola Solebo, Jugnoo S. Rahi

**Affiliations:** 1 University College London Great Ormond Street Institute of Child Health, 30 Guilford Street, London WC1N 1EH; 2 Great Ormond Street Hospital for Children NHS Ormond Street Hospital for Children NHS Foundation Trust, London, UK; 3 Ulverscroft Vision Research Group, UCL Great Ormond Street Institute of Child Health, University College London, UK; 4 Moorfields NIHR Biomedical Research Centre, London, UK; 5 UCL Institute of Ophthalmology, London, UK

**Keywords:** childhood, visual impairment, eye disease, economic burden, child health

## Abstract

**Background:**

Evidence on the socioeconomic burden associated with childhood visual impairment and blindness (VI/SVIBL) is needed to inform economic evaluations of existing and emerging interventions aimed at protecting or improving vision. This study aimed to evaluate the quantity and quality of literature on resource use and/or costs associated with childhood VI/SVIBL disorders.

**Methods:**

PUBMED, Web of Science (Ovid), the NHS Economic Evaluation Database and grey literature were searched in November 2020. The PUBMED search was rerun in February 2022. Original articles reporting unique estimates of resource use or cost data on conditions resulting in bilateral VI/SVIBL were eligible for data extraction. Quality assessment (QA) was undertaken using the Drummond checklist adapted for COI studies.

**Results:**

We identified 31 eligible articles, 27 from the peer-reviewed literature and 4 from the grey literature. 2 reported on resource use and 29 on costs. Cerebral visual impairment (CVI) and optic nerve disorders were not examined in any included studies, whereas ROP was the most frequently examined condition. The quality of studies varied, with economic evaluations (EE) having higher mean QA scores (82%) compared to cost-of-illness (COI) studies (77%). Deficiencies in reporting were seen particularly in the clinical definitions of conditions in EEs and a lacked of discounting and sensitivity analyses in COI studies.

**Conclusions:**

There is sparse literature on resource use or costs associated with childhood VI disorders. The first step in addressing this important evidence gap is to ensure core VI outcomes are measured in future randomised control trials of interventions as well as cohort studies and are reported as a discrete health outcome.

## 1 Introduction

Visual impairment, severe visual impairment and blindness (VI/SVI/BL) is permanent, bilateral visual disability which occurs in 1 per 1000 children in the United Kingdom (UK)[1]. Childhood VI/SVI/BL is caused by a constellation of rare disorders. For most children in this setting, onset is in the first year of life and is associated with non-ophthalmic multi-morbidity requiring multi-disciplinary specialist clinical management.^1^ Whilst childhood VI/SVI/BL is uncommon compared to the prevalence of adult-onset visual disability, the impact is disproportionately magnified by chronic co-morbid conditions, the early impact on development and activities of daily life and the cumulative years lived with impaired vision.

Effective and equitable planning and provision of specialist support, care and education is dependent on information on the socioeconomic impact of visual disability on the child, family, health system and wider society. Understanding the economic impact of childhood visual impairment is important for government decision makers to influence health policy, planning and delivery of key child health and specialist eye services. Economic or cost data is particularly important for the new approval of novel treatments for provision in the National Health Service (NHS) in the UK, where the National Institute for Health and Care Excellence (NICE) mandates the submission of persuasive health economic evidence, specifying preferred methods[2], alongside efficacy and safety data.

The Lancet Global Health Commission on Global Eye Health recently carried out a systematic review of health economic literature of eye health interventions for all ages [3][4], but it did not report data specifically in children. This impairs our understanding of the health economic landscape in blinding disorders occurring in childhood, an area which remains an important gap in the literature. We report here a systematic literature review to describe the quantity and quality of health economic literature pertaining to childhood visual disability or contributing disorders.

## 2 Methods

We systematically reviewed cost of illness and economic evaluation studies of disorders that cause VI/SVI/BL in childhood in high income settings. This review is reported in line with the Preferred Reporting Items for Systematic review and Meta-Analysis (PRISMA) guidelines [5,6].

### 2.1 Data sources and search strategy

PUBMED, Web of Science (Ovid) and the NHS Economic Evaluation Database (NHS EED, including the CRD, DARE and HTA databases) were searched for peer-reviewed articles on 17 November 2020. Additional articles were added through ’snowballing’ of bibliographies and reference lists of eligible articles. A search was also carried out in the Google™ engine as well as in Google scholar™ to identify additional peer-reviewed articles and grey literature, including pertinent cost of illness reports by governmental and non-governmental organisations such as supranational bodies or eye charities. The search strategy used for publications in scientific databases combined key words related to visual impairment, blindness and eye disorders, cost and cost of illness synonyms combined with an amended child filter[7]. The full search strategy is presented in Figure S1 alongside the grey literature search strategy. The PUBMED search was rerun on 21 February 2022 to include new studies published since the original search.

The protocol for this review is registered on the Research Registry (reviewregistry1378).

### 2.2 Inclusion criteria

We included any original research article that reported the economic burden, defined as a measure of resource use and/or associated costs, for the diagnosis, treatment, or prevention of a disorder resulting in childhood visual impairment or blindness, defined as any ocular or cerebral disorder contributing to the incidence of childhood VI/SVIBL[1] The disorders resulting in childhood VI/SVI/BL were based on those reported in the only nationally representative epidemiological study of full spectrum childhood VI/SVI/BL in a high income setting[1]. To be included, articles had to report a unique estimate of resource use or costs in children (which we defined as the mean or median age of the included population of 18 years or less). There was no restriction on country or language.

### 2.3 Screening & Data extraction

References were exported into the Covidence systematic review software[8] for duplicate removal and for screening. Abstracts were screened independently by two reviewers, and three reviewers screened full texts and extracted data (two reviewers screening 50% articles each and one reviewer screening 100% of articles). Conflicts were addressed through group discussion until a unanimous decision was reached. A standardised data extraction form was used to record data items comprising population specific information (Table S1). Included studies are grouped by anatomical ophthalmic site, following the WHO classification of causes of visual impairment [9].

### 2.4 Methodological (quality) assessment

The checklist published by Molinier et al[10] was used to assess the methodological quality of included studies. This 11-point checklist adapted from the Drummond checklist[11] for COI studies was also used for economic evaluations (Table S2) in the absence of an international consensus on a quality assessment tool for economic studies. Each question in the checklist is of equal weight, and scored as Yes, No or Partially or not specified. The total score was the sum of across all 11 items.

The same checklist was applied to grey literature to provide a framework for quality assessment in the absence of a formal tool assessing for grey literature reports presenting costs.

### 2.5 Synthesis & Analysis

Due to the heterogeneity of included studies (differences in definitions of ophthalmic disorders, cost categories included, resource valuation and geographic setting) we did not carry out a meta-analysis in order to present pooled cost estimates for each ophthalmic condition. Instead we present a narrative review of the studies, following the reporting guidelines for narrative synthesis for systematic reviews without meta-analysis (SWIM)[12].

The main cost measure is presented alongside the cost estimate, most of which are presented as annual, per person costs for each ophthalmic condition, stratified by cost category. Studies reporting costs of visual impairment as an outcome (all-cause VI/SVI/BL) are presented separately, including the level of visual acuity authors used to define visual impairment. Cost estimates were standardised to allow for comparison of costs between studies using different currencies. Standardisation involved first converting the main cost estimate to a base year of 2020 by multiplying by the World Bank consumer price index[13]; a Gross Domestic Product [GDP] deflator) adjusting for inflation, then the Purchasing Power Parity index[14] was applied to convert the reported currency into US dollars (USD).[14]

## 3 Results

**Figure 1:**
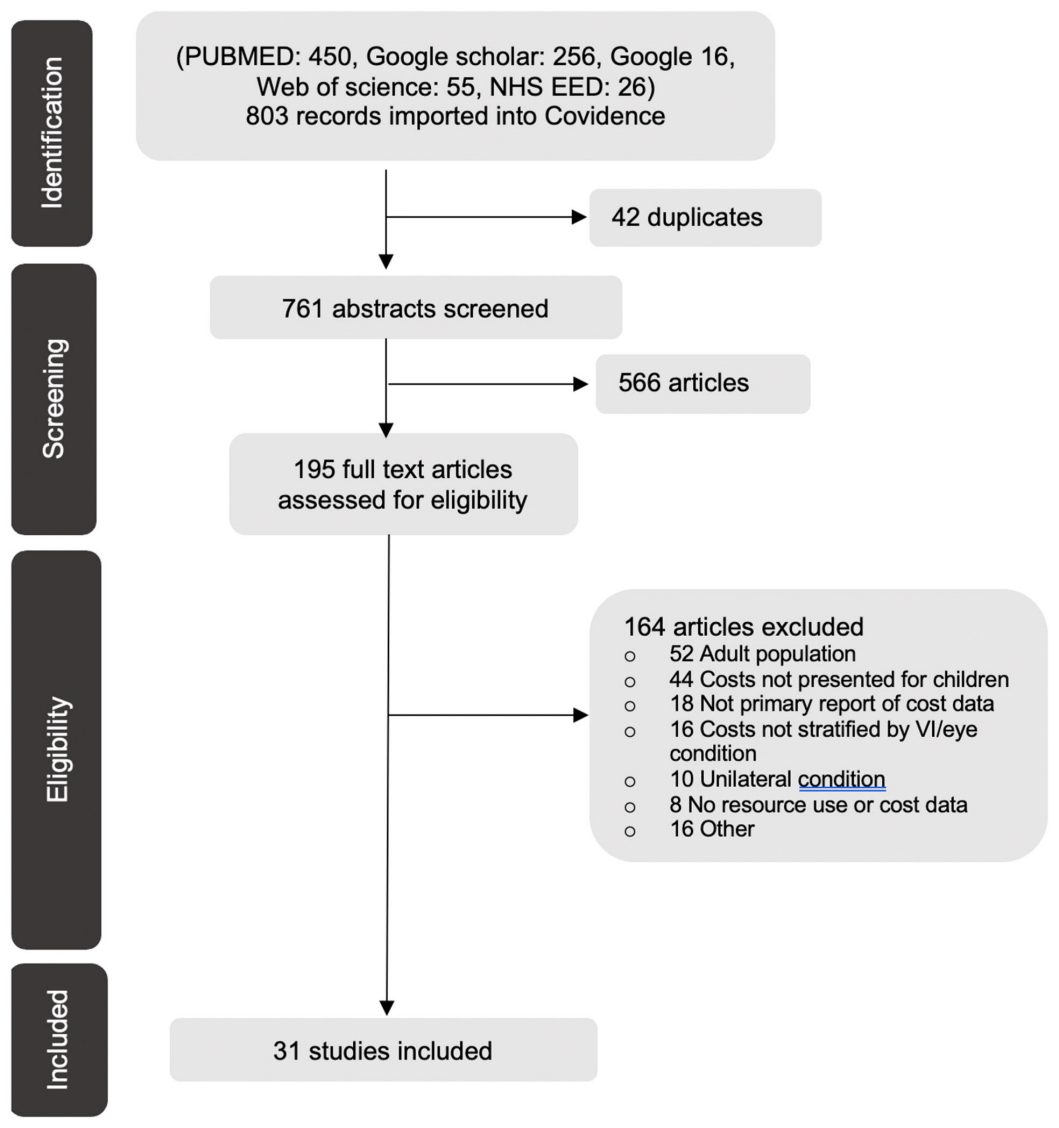
PRISMA flow chat of screened and included studies

Our search identified 803 abstracts. Following screening, 31 articles (27 articles from peer-reviewed journals and 4 grey literature papers[15–18]met our inclusion criteria (Figure 1). The full report of one grey literature report could not be found[17], so the summary version of the report was used for data extraction and quality assessment.

### 3.1 Data Sources

The majority of included economic evaluations used data collected in the context of randomised clinical trials and follow up of the original trials (66%, 10/15). Economic evaluations that did not employ resource use and costs data generated from randomised controlled trials, used published data from the literature (*n*=2 [19,20]) [19,20], expert opinion from ophthalmologists (*n*=1 [21]) and from hospital records (*n*=2 [20,22,23]).[19,20]

For COI studies, resource use data was collected from a variety of sources from both secondary data sources such as health insurance claims databases (*n*=2 [18,24]), hospital databases (*n*=3 [24–26]), national figures, e.g. Gross National Product (GNP) per capita (*n*=1) and primary sources including cost questionnaires to families (based on patient recall) (*n*=4 [25–28]). For the 3 case studies [29–31]), one employed a daily cost diary completed by the patient over 1 year[30], one used a database from a national resource centre[29], and one used cost estimates from government sources (but these sources were poorly reported[31]).

Grey literature reports used aggregated health system costs or expenditures from national health authorities, surveys and health insurance claims databases as sources of cost data. The lack of national incidence rates of childhood specific visual impairment was evident. Only one report estimated the national burden in the US though this was done in the absence of national rates in children under 12 years.[32]

### 3.2 Study design

Table 1 summarises the study design, age range, sample size and country of included studies. Of the peer-reviewed articles (*n*=27), 15 were economic evaluations (56%); 9 were cost of illness studies and 3 [29–31]were described as ‘case studies’ that measured or estimated resource use or costs in a small number of children (study sizes ranging from n=3 to *n*=869). Of the 15 economic evaluations, 6 were cost-utility analyses (CUA), 4 were cost-effectiveness analyses (CEA), 3 were cost-minimization analyses (CMA) [20,21,23], 1 reported outcomes of both a CUA & cost-benefit analysis (CBA)[33] and 1 was a budgetary impact analysis [19]. Almost all of these economic evaluations used decision analytic models (14/15), of which 10 used decision tree analysis and 4 used Markov models. 1 study did not specify the ‘cost model’ used[34].

The 9 cost of illness studies were of various designs including use of synthetic cohorts (Table 1), whilst the additional 4 grey literature reports (3 reports commissioned by the third sector [17,18,35], one book chapter[15]) were cost of illness studies using secondary data analysis.

### 3.3 Resource valuation

Costs of medical resource use were acquired from a range of sources comprising reimbursement rates and expenditure data from health insurance providers; national reference hospital costs or charges, or specific hospital department costs; published literature, including published medical association reported statistics; and collection of receipts from interviewed families. Productivity losses were calculated using national average salaries or earnings[15,28,35–38] or GDP[26,39,40] or GNP per capita[41].

#### Methodological quality

The average quality assessment score across all studies was 77% (median score 79%) and scores ranged from 39% to 100%. Quality assessment scores varied by study type, with economic evaluations scoring the highest mean scores at 82%, 77% for cost-of-illness studies, and 56% for case studies. The mean score for 3 of the 4 grey literature reports was 73%.

For economic evaluations, items that had the poorest scores were clear definition of the illness/condition’ (8/16 articles were only partially defined), ‘discounting of costs’ (absent in 7/16) and ‘description of epidemiological sources’ (partial description only in 5/16). For cost of illness studies, sensitivity analyses were not carried out in the majority of studies (6/8), and the data sources of resource use and cost values were not thoroughly or clearly described in 4/8 articles and discounting of costs only occurred in half of COI studies (4/8). For case studies, both the sources of resource use data as well as the appropriate method of assessment required improvements.

Notably, the reporting of a clear definition of the ophthalmic condition in peer-reviewed articles, occrred in a slightly higher proportion of non-ophthalmic journals compared to ophthalmic journals; 58% vs 53% were reported clearly and 30% vs 40% were partially reported.

##### Analytic Perspective

The societal perspective was the most common perspective used across studies (*n*=14), followed by the perspective of the payer (including third party payer) (*n*=7), healthcare system (*n*=6), patient perspective (*n*=2) [28,30], and one article used ‘Other’ perspective which was from the Government sector.[42] One article did not report the perspective used[41].

#### Cost outcomes

Cost outcome measures reported in studies were heterogeneous and varied by study type (Table 3.). For economic evaluations, the most common cost outcome was cost per quality adjusted life year (QALY) (9/16), followed by cost savings per child (4/16), incremental net monetary benefit (INMB, 2/16), and incremental cost per treated infant (1/16).

COI studies reported more heterogeneous cost measures, which reflected the specific research question being addressed. Lifetime costs were estimated in 2 studies, one study measured the cost difference between treatments, one study presented costs before, during and after treatment; 2 studies calculated eye/vision specific costs, 1 reporting the mean annual cost of eye involvement in children with Juvenile Idiopathic Arthritis (JIA)[25] and 1 study that reported the marginal annual health cost of visual disturbance or blindness[43] in the fifth year of life in children born premature[43].

Only 6 (all peer-reviewed) of the 31 studies stratified cost or resource use estimates by relevant clinical subgroups or risk factors. 4 articles stratified by patient risk factors, included 3 ROP studies using birthweight[19,42,44], and 1 study in preterm infants stratified by gestational age[43]. 1 article stratified costs by disease severity (JIA uveitis [25]) and only 1 study stratified by the presence of comorbidities [43]. 1 study stratified costs by ophthalmic disease subtype, which separated costs for primary childhood glaucoma and secondary childhood glaucoma[45].

##### Cost categories

20 studies estimated healthcare costs, 2 estimated non-health costs, 15 estimated indirect costs however only 9 studies presented total indirect costs separately. Of the 15 studies that estimated indirect cost, 4 only measured productivity losses (lost wages or income) associated with caregiving (informal care), 3 only measured productivity losses associated with visual disability (lost wages as an adult) and 6 studies measured both. 3 studies did not specify the type of productivity losses measured. Other indirect costs included were [18,32] deadweight losses (taxes and transfers)[18,35] and premature mortality[15]. 9 studies included costs associated with special education due to VI.

##### Distribution of ophthalmic conditions

Specific ophthalmic conditions were investigated in the majority of articles (22/31, 71%), whilst 9 investigated all cause VI/SVI/BL (including all four of the grey literature articles).

**Figure 2:**
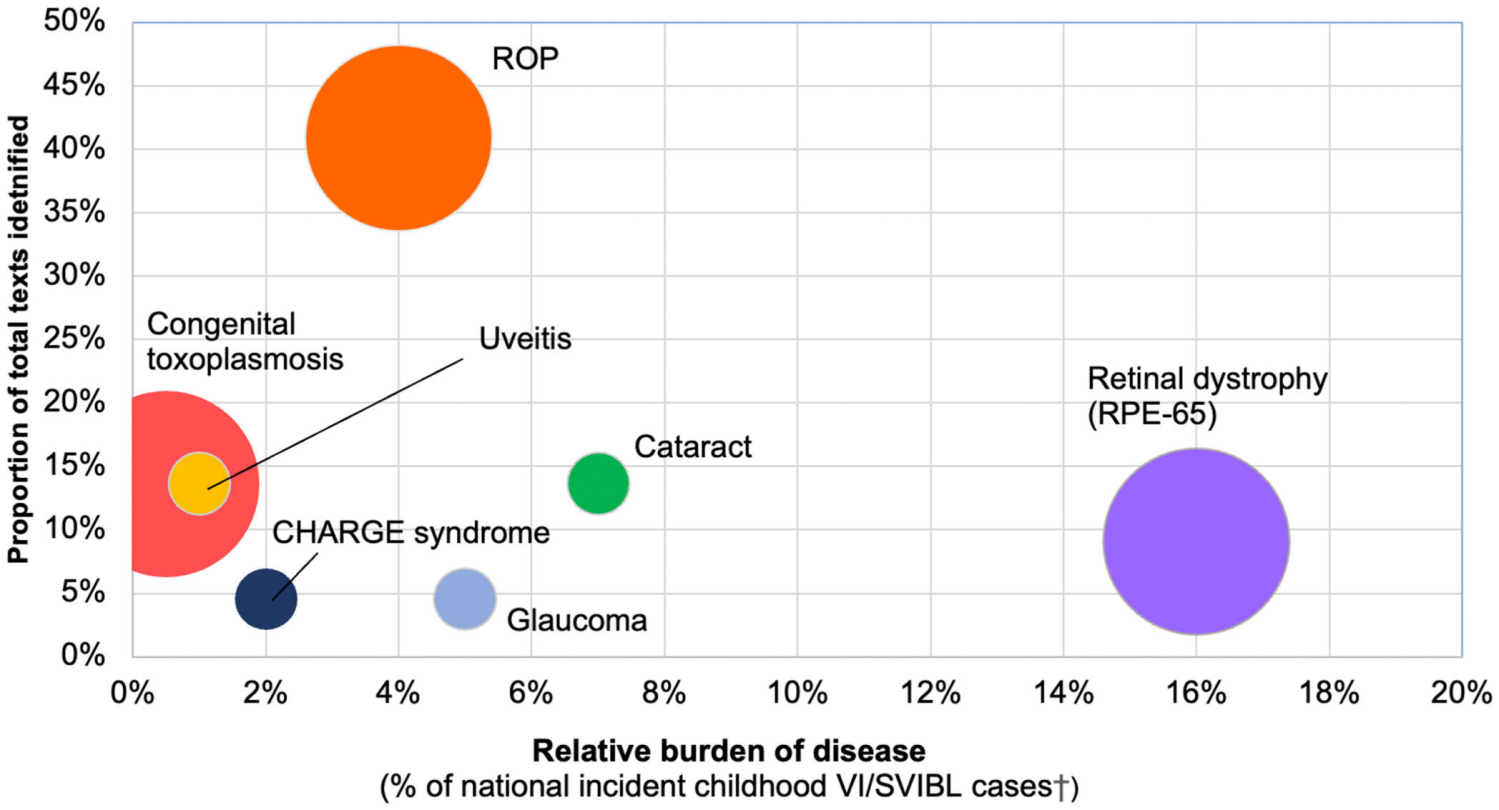
Ophthalmic conditions examined in the health economic literature and their relative contribution to burden of childwood visual impairment in high incoming settings †calculated as number cases from ophthalmic disorder/total cases in BCVIS2^1^ *size of each bubble is proportionate to the relative burden of each disorder in high income countries. Data used was from Table 1.6 Taylor and Hoyt textbook^2^

Figure 2 plots the number of articles identified for each ophthalmic condition represented in the economic literature against the relative burden of each ophthalmic condition in the U.K[1]. The size of the bubbles is proportional to the relative burden across all high-income settings, based on global burden of disease data. The dimension of relative global burden was added to allow comparison of U.K data to other similar settings. Figure 2 shows that retinal conditions were most commonly studied (*n*=14), with retinopathy of prematurity (ROP) the most common specific condition studied [19,26,34,39,40,42,46–48] (9/22, 41%) and well represented in the literature compared to the relative burden of childhood VI/SVI/BL (4%). Three articles examined bilateral congenital or childhood cataract [22,24,28](14%). However, whilst the incidence of ROP and cataract is low in HIC, each is a significant cause of childhood VI/SVI/BL globally due to the high burden in low/middle income countries (LMIC). RPE-65 mediated retinal dystrophy (2/22, 9%), represented the highest burden of any included individual condition (all retinal dystrophies together caused 16% of annual childhood VI). Conversely, childhood uveitis[21,25,49] and congenital toxoplasmosis[20,23,36] (each contributing 14% of articles) were also well represented in the literature in contrast to the relative (proportional) burden. Finally, two articles examined disorders of the globe; 1 in childhood glaucoma (primary and secondary[37]) and 1 in CHARGE syndrome[29] (coloboma, microphthalmos).

No studies examined resource use or costs of vision loss resulting from injury or insult to the visual pathways or cortex (referred to as cerebral visual impairment; CVI), the leading cause of childhood visual impairment in high income settings[1], and a significant cause globally[50]. Disorders of the optic nerve and CVI were not represented in the health economic literature and are thus important ophthalmic sites missing from Figure 2 and why proportions do not total to 100%. One study did estimate the marginal healthcare cost of visual comorbidities and blindness in children born preterm, but did not specify what causative disorders were included, so the cost associated specifically with CVI or ROP could not be distinguished.

##### Time horizon

For economic evaluations, there were generally two groups of studies, those with a very short time horizon (the period over which health outcomes and costs are calculated), between 8 weeks and 18 months (*n*=6) and those adopting a lifetime horizon (*n*=9). For COI studies the time horizon was more heterogeneous and varied between 1 year (*n*=2), 4 years (*n*=2) and a lifetime horizon (*n*=1).

##### Sensitivity analysis

93% of economic evaluations (14/15) carried out some form of sensitivity analysis. Deterministic (DSA) as well as probabilistic sensitivity analyses (e.g. Monte Carlo simulation models), were carried out in 7 articles, and a further 7 only carried out DSA (e.g. one or two way sensitivity analyses) (Table 2). This is compared to sensitivity analyses being carried out in only 2/9 COI studies, all of which were limited to one way sensitivity analyses. Grey literature reports were less likely to incorporate sensitivity analysis (2/4) than the peer-reviewed literature, only one report used univariate sensitivity analysis (varied the discount rate only) and another carried out robust sensitivity analysis using both one away and probabilistic methods.

## 4 Discussion

In this systematic review of the literature assessing resource use and costs specifically of childhood disorders causing visual disability, we report a scarcity of representative, high quality studies, and heterogeneity of disorder definition, study populations, study metrics and analytic approaches. Retinopathy of prematurity was the most frequent single condition examined, whereas cerebral visual impairment was not represented at all in the evidence base.

This literature review carried out a broad and inclusive systematic search strategy of peer-reviewed literature of childhood ophthalmic conditions that can lead to significant bilateral vision impairment, without restrictions of year of publication, geographical setting or language. Our review also included grey literature, which is an important source for patient information and for advocacy through the third sector. Grey literature can negate the influence of publication bias and help to provide information on the context of healthcare interventions[51]. We present key extracted data from studies by anatomical site, using the WHO international classification, for ease of interpretation by clinicians, decision makers and researchers.

Study limitations for this review include, the search strategy, which focussed on resource use or costs of bilateral childhood visual disability in high income settings, and thus excluded articles examining uncorrected refractive error (which carries a significant burden in low income settings) and unilateral conditions such as strabismus (squint) and unilateral cataract. Secondly, due to the significant methodological heterogeneity between studies, particularly the differing definitions of visual impairment used, a meta-analysis could not be carried out. However, this aligns with Cochrane guidance on meta-analysis of cost data, that advises ‘extreme caution’ when considering a meta-analysis for reviews[52] due to the variation in resource use and costs across settings.

Although the impact of visual disability on the quality of life of the visually impaired child is known to be significant, representative data sources on children and young people remain scarce[53], whilst studies in adult populations are increasing[54]. It is promising however, that there is increasing recognition of the importance of child-specific approaches to measuring health related quality of life and assessing interventions for paediatric populations[55] which will hopefully lead to more economic evaluations in this area.

In terms of economic evaluations (EEs), the availability of robust treatment efficacy and resource use data that is collected alongside randomised controlled trials (RCTs) clearly drives the quantity of EEs of blinding childhood disorders, due to the availability of effective treatments and development of novel therapies. Thus, ophthalmic disorders for which treatments are available and have been assessed in RCTs were most frequently studied in the literature, including ROP, uveitis and RPE-65 mediated retinal dystrophy. The latter two conditions reflect the recent availability of new disease-modifying treatments that have been assessed in RCTs. For JIA associated uveitis, the use of a tumour necrosis factor (TNF-α) monoclonal antibody (adalimumab) has been recently shown to be clinically effective in children in the SYCAMORE trial[56], which triggered a cost-utility analysis to assess cost-effectiveness of its use[57]. Similarly, the evidence of clinical effectiveness of a novel virus-vector based targeted gene therapy (voretigene neparvovec) for RPE-65 mediated retinal dystrophy in a phase III clinical trial[58] precipitated two economic evaluations included in this study, one in the U.S.[59] and one in the U.K.[60] both using data generated from the trial.

Only one study focussed on an ‘untreatable' cause of childhood vision loss, a case study measuring healthcare resource use of children with CHARGE syndrome[29]. Whilst ‘untreatable’ conditions lack interventions aimed at preventing or ‘curing’ disease, complex interventions aimed at preventing the avoidable lifelong burden of reduced vision have been shown to have a positive impact on outcomes[61].

The notable absence of studies on the economic impact of cerebral visual impairment (CVI) represents a significant gap in the literature, as CVI is one of the leading causes of childhood VI in high-income settings[62,63] including in the U.K. where it is the responsible for almost half (48% [1]) of the burden of childhood visual impairment. This is particularly worrying given the incidence of CVI is likely to increase globally as provision and quality of neonatal care improves and the consequent survival of preterm babies with neurodevelopmental sequelae increases. The absence of CVI studies in the health economic literature reflects the nascence of RCT evidence on both preventative interventions (aimed at the underlying cause of CVI, such as hypoxic ischaemic encephalopathy (HIE)) and interventions focussing on CVI as a functional outcome. A recent scoping review[64] examined the latter type of CVI intervention identifying only 3 RCTs and reported key limitations of the published literature including inconsistency of outcome measures, low quality evidence and small sample size. Published trials of neuroprotective interventions targeted at the underlying causes of CVI, such as HIE, have been inconsistent in their inclusion of visual related outcome measures. For example, only 7 of the 11 trials identified in the Cochrane review of hypothermia reported including visual outcomes[65], with the remaining trials only including visual outcomes subsumed within a composite measure of neurodevelopmental or severe disability or were not included at all [66–69].

This review therefore highlights that disorders that significant causes of the burden of childhood VI are missing from the health economic literature. Thus increased attention is needed on CVI and ‘untreatable’ disorders for which tertiary prevention interventions (aimed at reducing the negative impact of established disability) exist, such as habilitation training and low vision aids. As research into neonatal neuroprotective interventions which could prevent CVI expands, it is crucial that visual outcomes are included as core outcomes measures of future trials so these data are available for use in cost-effectiveness models. Childhood visual disability is recognised as a ‘sentinel’ child health event,[1] and as such, vision should always be a discrete outcome measure in randomised controlled trials and cohort studies of preterm infants, and ophthalmic and vision outcomes should be reported explicitly so that decision models in economic evaluations of interventions can be undertaken.

Aside from the current review, a recent systematic literature review delivered as part of the Lancet Global Health Commission on Global Eye Health [70] evaluated the societal economic impact of the seven leading causes of vision impairment across all ages globally, so included uncorrected refractive error and corneal opacity. This review by Marques et al, identified only 4 peer-reviewed articles involving children and recommended the development of a ‘reference case’ for eye health; a reference document outlining costing methods to better support decision making[70]. Our review supports and extends this recommendation, with findings that highlight the importance of inclusion of specific data items to reduce heterogeneity in methods and resulting cost measures, particularly in cost of illness studies. A second recent review examining economic evaluations in ROP[71] in the U.S., Canada and U.K., included screening studies which the current review excluded. The authors found limited literature (only 9 papers) and found that whilst interventions were highly cost-effective, methodological improvements in reporting of some items was necessary.

Half of the COI and EE studies included in our review used a lifetime time horizon, modelling costs and outcomes across the lifecourse, even though there is a recognised lack of “valid” longitudinal (incidence based) data for conditions during childhood and into adulthood. This lack of long term data starting from childhood is a recognised challenge in constructing lifetime models of disease and economic impact[72]. Representative longitudinal data on VI conditions of childhood is therefore needed to provide robust inputs to health economic models and improve the accuracy of findings. In particular for countries with national hospital administrative databases, the routinely collected primary and secondary care resource use data could be harnessed to fill in the gaps in our understanding of specific ophthalmic disorders that affect children, capitalising on the national coverage of such datasets.

In terms of quality, this review highlights the need for better reporting of key components of COI and EEs in childhood VI. Economic evaluations require clear reporting of the definitions of ophthalmic disorders examined and detailed descriptions and transparency in reporting of epidemiological data sources. Both EEs and COIs should incorporate discounting in cost analyses with longer time horizons or report the reasoning for not applying a discount rate to costs.

COI studies should include sensitivity analyses to assess the robustness of models used alongside more detailed descriptions of epidemiologic data sources. The quality of grey literature reports could be improved substantially, with more transparent and descriptive reporting of data sources, and explicit statement of the rationale behind the assumptions used. Finally, many studies of childhood VI did not state the visual acuity thresholds used to define severity of visual disability, therefore a final recommendation is for future studies to explicitly state how impairment is classified and the rationale for choosing definitions other than the WHO taxonomy[73].

## Conclusion

Our review demonstrates the significant evidence gap in understanding of the health economic burden of childhood vision impairment and identifies the methodological issues that need to be addressed as well as the opportunities to address this evidence gap. This lack of ‘visibility’ of the health economic impact of childhood visual disability is an important factor in the lower priority given to children in vision and public health policies globally.

## Supplementary Material

Supplementary information

## Figures and Tables

**Table 1:** Summary of included studies, by ophthalmic site and ophthalmic condition examined

First Author	Year of pub	Eye condition/ VI level	Country(ies)	Paediatric age range*	Sample size	Study type	Study design
**CHARGE syndrome**		***WHOLE GLOBE AND ANTERIOR SEGMENT***					
Anderzen-Carlsson^29^	2014	CHARGE syndrome: coloboma, microphthalmos, strabismus	Sweden	1-20 years	5	Case study	Case study of healthcare use in first year of life
**Glaucoma**							
Liu^37^	2016	Childhood glaucoma: primary glaucoma (PCG) and secondary glaucoma(SCG)	USA	0-21 years old	23	COI - Retrospective, single centre	Retrospective study of all children who presented with glaucoma to single tertiary hospital in 15 year time period - examined costs up till 4 years since diagnosis
***LENS (Cataract)***							
Cernat^22^	2022	Cataract (Bilateral)	Canada	0-2 years at time of surgery	53 children	Economic evaluation	Cost-effectiveness analysis using decision tree model to assess C/E of ISBCS (immediate sequential bilateral cataract) compared to DSBCS (delayed sequential bilateral cataract surgery)
Dave^24^	2010	Congenital cataract (Bilateral)	USA	< 6 months old	27	COI - Retrospective, single centre	Retrospective cost (and outcomes) comparison study of simultaneous vs sequential cataract surgery carried out by one surgeon in one hospital. Children followed up for on average 2.5 or 5 years (depending on surgery). 'Cohort study'
Wang^28^	2018	Bilateral developmental non-traumatic paediatric cataract	China & India	0-10 years old	181	COI - Prospective, multi-centre, cross-country	Cost of illness study - enrolled consecutive families for 1 year with bilateral cataract visiting 2 hospital sites in China and India.
***UVEA (Uveitis)***							
Hughes^49^	2018	JIA Uveitis	UK	2-18 years old	90 participants in SYCAMORE trial	Economic evaluation	Cost-effectiveness analysis of adalimumab +methotrexate vs methotrexate alone (Markov model)
Minden^25^	2009	JIA Uveitis	Germany	2-18 years old	54 children with JIA uveitis (out of total of 369 children with JIA)	COI	Cross-sectional cost of illness study
Noble^21^	2008	Nongranulomatous anterior uveitis	Canada	“child” no years defined	N/A	Economic evaluation	Cost minimization analysis comparing costs of 'investigating' a child with AU per current practice guidelines compared to published evidence based guidelines.
***RETINA***							
**Congenital toxoplasmosis**							
Prusa^23^	2017	congenital toxoplasmosis (retinochoroiditis, blindness)	Austria	‘congenital’ toxoplasmosis	N/A incidence rates used	Economic evaluation	Decision tree model (prenatal serological screening program and treatment vs no screening program). Also calculated the budget impact in Austria
Roberts (&Frenkel)^36^	1990	congenital toxoplasmosis	USA	?0-18 years old	N/A used incidence rates	COI - evidence synthesis	Cost of illness study: Estimated lifetime costs, including incomes losses using the human capital calculation and other costs taken from the literature based on assumptions of healthcare for children with each type of impairment (mild/moderate/severe mental retardation, blind, deaf)
Stillwaggon	2011	congenital toxoplasmosis	USA	Not reported	N/A used incidence rates	Economic evaluation	Cost minimization analysis using decision tree model - comparing universal maternal serological screening and prenatal treatment (psyrimethamine, sulfadiazine, folinic acid etc) vs no systematic screening.
**Retinal dystrophy**							
Viriato^60^	2020	Inherited retinal dystrophy (biallelic, RPE-65 mediated)	UK	mean age of trial participants was 15.1 y, median: 14y (range 4-44 year)	31 (from trial) - includes some adults	Economic evaluation	Cost Utility analysis using a Markov model to estimate incremental cost per QALY of Voretigene neparvovec (VN) compared to best supportive care (BC)
Zimmermann^59^	2019	Inherited retinal disease (biallelic RPE-65 mediated)	USA	mean age in trial: 15 years	not stated	Economic evaluation	Cost Utility Analysis using a 2 state Markov model comparing Voretigene neparvovec (VN) to standard care - modelled over lifetime
**Retinopathy of prematurity**							
Brown^46^	1999	ROP - Threshold	USA	Infants; average age at treatment was 3 months of age	291 infants from CRYO-ROP trial at 3.5y after randomisation	Economic evaluation	Cost effectiveness analysis using a decision tree model (3 armed). Treatment: cryotherapy vs natural course of disease & laser photocoagulation therapy vs natural course
_Dave_^26^	2012	ROP associated blindness (bilateral)	Peru	neonates	synthetic cohort: 400 children treated over 20 years	Economic evaluation	Cost-effectiveness analysis using synthetic cohort also estimates the generational savings of early treatment of ROP. Screening & treatment (diode laser compared to no treatment ‘natural course of disease’)
Dunbar^34^	2009	ROP (threshold)	USA	Infants	515 infants screened	Economic evaluation	Cost utility Analysis (screening and treatment of ROP - cryotherapy vs laser therapy)
Jackson^47^	2008	ROP (Threshold): unilateral and bilateral	USA	Infants stratified into <1.5kg & <1.251kg)	Not stated	Economic evaluation	Cost utility analysis: decision tree model, comparing telemedicine (image captured by trained neonatal personnel) vs standard ophthalmoscopy (for ROP management) including laser treatment
Javitt^42^	1993	ROP (threshold)	USA	Infants (preterm)	modelled birth cohort of 28321 infants with birthweights between 500g-1.249kg	Economic evaluation	Cost utility analysis: of screening and cryotherapy for threshold ROP using microsimulation model. Screening & treatment (cryotherapy). 3 difference frequencies of screening compared in model: weekly, biweekly and monthly
Kamholz^48^	2007	ROP (high risk pre-threshold)	USA	Infants	Not stated	Economic evaluation	Cost-effectiveness analysis using a decision tree model. Screening & treatment: early treatment of ROP vs conventional management
Naguib^39^	2019	ROP related blindness	Philippines	3-28 years (number of children <18y not presented)	N/A used incidence rates	Economic evaluation	Cost-effectiveness analysis and IMB calculated using decision tree model: for ideal national ROP programme (universal screening and treatment pan retinal photocoagulation - 30wks or less &1.5kg or less) compared to current screening (32wks or less and BW <1.5kg)
Rothschild^40^	2016	ROP	USA & Mexico	Infants/neonates	95 (52 children in Atlanta and 43 in Mexico City) completed economic (EcROP) survey	Economic evaluation	Cost utility analysis and CBA using decision tree model - comparing 1) current levels of screening and treatment to 2) ideal national screening programme (Bevacizumab or ranibizumab) program vs current practice with 100% screening coverage and appropriate management and treatment of all infants with ROP.
Zin^19^	2014	ROP	Brazil	newborn infants	869 preterm infants	Economic evaluation	3 economic approaches used: 1) cost analysis 2) incremental cost analysis 3) budget impact analysis of incorporating the detection and treatment of ROP screening and treatment (laser) into neonatal care services.
***ALL CAUSE VISION LOSS***							
Australia Save Sight Institute^35^	2016	All childhood eye conditions that result in 'blindness or severe vision impairment' - including unilateral and correctable refractive error	Australia	0-17 years old	used prevalence rates	COI - evidence synthesis	Cost of illness study using evidence synthesis from published literature, survey and requested aggregate health system expenditure data.
Guide Dogs^17^	2003	“congenital visual impairment, e.g. Retinitis pigmentosa”, otherwise undefined	UK	adolescence’ - otherwise, not specified	Not stated	COI - evidence synthesis *very little information found as full report could not be found*	No information provided on methodology
Honeycutt^15^	2003	Vision impairment - undefined	USA	0-17 years old	N/A used prevalence rates	COI - evidence synthesis	Cost of illness - estimated lifetimes costs using incident cohort (children born in 2000). Used number of children expected to survive at each age group
Korvenranta^27^	2010	Blindness & visual disturbances of very premature infants	Finland	Preterm infants (<32 weeks or birthweight <1501g) at the 5th year during life	588 very preterm infants (175 control subjects)	COI - Prospective, cohort	National cohort of preterm babies identified at all Finnish hospitals with (level 2 or 3) NICUs
O’Connor^30^	2008	2 children with bilateral moderate visual impairment (VI), 1 YP bilateral blind, one child with mild vision loss (6/7.5, 6/9)	Australia	10 -19 years old	3 (children with VI or BL), 1 (‘mild’ vision loss)	Case study	Exploratory case study - prospective. Diary study of children with 3 different levels of vision and 3 different age groups:
Shamanna^41^	1998	Childhood blindness - undefined	India	mean age of 8 used for children (but no original data collected for paediatric population)	N/A	COI - evidence synthesis	Cost of illness' using cost and GNP per capita - using data from the literature only (no original data collected)
Wittenborn^18^	2013	VI (all cause mild (>20/40), moderate vision impairment (>20/80) and blindness (20/200))	USA	0-17 years old	N/A prevalence rate used	COI - evidence synthesis	Cost of illness, estimating total costs of population (0-17y and 18-40y) in the USA attributable to eye disorders
Wittenborn^38^	2013	All diagnosed eye disorders, undiagnosed self-reported vision loss and vision correction	USA	0-17 years old	N/A prevalence rate used	COI - evidence synthesis	Cost of illness, estimating total costs of population (0-17y and 18-40y) in the USA attributable to eye disorders, undiagnosed vision loss and vision correction
Wright^31^	2000	“Congenital visual impairment” - undefined	Australia	<16 years old (case study 3; 'school student')	Case study of 1 student	Case study	3 'case studies' for difference age ranges in a typical case. compiling government assistance & other financial costs in 1 year

* or mean/median age if range not reported

**Table 2:** Detailed description of resource valuation, cost components and sensitivity analysis of included studies

First Author	Resource valuation	Cost components (resource use items)	T rial data used	Sensitivity analysis
***WHOLE GLOBE AND ANTERIOR SEGMENT***				
**CHARGE syndrome**				
Anderzen-Carlsson^29^	N/A - study only reported healthcare use (contacts)	Medical costs *(hospital care, diagnostic procedures, pharmacological treatment, surgical treatment, multidisciplinary care)*	n/a	N/A
**Glaucoma**				
_Liu_^37^	Hospital specific charges used - taken from national NHCUP 2015 published charge to cost ratios. National average hourly wage (in 2013) used to calculate indirect costs.	Direct medical costs *(EUA + anaesthesia, surgeries, glaucoma related office and emergency visits, topical ocular medications)*. Indirect costs *(average number of missed workdays by caregiver*)	n/a	No
***LENS (Cataract)***				
Cernat^22^	Unit hospital costs taken from Ministry of Health costing tool, physician fees taken from MoH schedule of benefits. Drug costs taken from national formulary.	Hospital costs (*surgery, physician visits, anaesthesia, drugs, EUA, glasses fitting*), parental out of pocket expenses *(parking fees, contact lenses, glasses*)	n/a	Yes: one way: varied key parameters including surgical costs, number of follow up hospital visits and perspective
_Dave_^24^	Reimbursement rates (Medicaid payments) using procedure codes (CPT-4) used for hospital and physician use	Medical costs included hospital cataract surgery/procedure *(cataract extraction, primary posterior capsulotomy and anterior vitrectomy*, *anaesthesia, drugs & supplies, Physician follow up*)	n/a	No
_Wang_^28^	Costs taken from receipts/invoices from family. Salary loss based on reported monthly salary of family	Direct and indirect costs, including *hospital treatment, transport, food, lodging, economic losses due to absence from work*	n/a	No
***UVEA (Uveitis)***				
Hughes^49^	Unit costs from standard NHS sources (national schedule of reference costs 2015-16) and PSSRU for community care services	*Hospitalisations, outpatient clinic visits & procedures, A&E admissions, GP consultations, medications*.	SYCAMORE	Yes: univariate, bivariate and probabilistic sensitivity analysis; Monte Carlo simulations (10,000 replications)
Minden^25^	Unit costs for hospital admission, therapy and drugs from national statutory health insurance statistics. Mean annual income from individuals (dependent work) taken from Federal Statistical Office.	Direct medical costs *(inpatient, outpatient, drugs, physician visits, devices and aids*), non-health care costs (*family out of pocket expenses, special equipment*. Indirect costs: parental time lost from work due to JIA related caregiving (productivity losses)	n/a	Yes: varied reimbursement rates of outpatient and inpatient stays
Noble^21^	Cost data from Ontario Ministry of Health schedule of benefits and province laboratories.	Cost of ‘investigating patient with uveitis’ - cost associated with diagnosis only.	n/a	Yes - Two way SA and PSA: Monte Carlo simulations
***RETINA***				
**Congenital toxoplasmosis**				
Prusa^23^	Costs taken from mean regional health insurance reports, special education costs & productivity costs taken from OECD	Hospital costs, *diagnostic testing (serology), non-medical costs, productivity costs (lost earnings) of parents (caregiving) and infant special education costs and also includes cost associated with infant death*	n/a	Yes: using incremental tornado diagram (varying all costs)
Roberts (& Frenkel^36^	Hospital costs were sourced from national hospital statistics (American Hospital Association). Productivity losses-used average weekly earnings reported by Bureau of Labour - estimated present value of infant’s life.	Medical cost assumptions included: *2 weeks of intensive care, 2 weeks in regular hospital room following severe illness as infant, annual eye examinations and an operation for strabismus*. Productivity losses (*assumed income losses for impaired survivors to be 70% of lifetime income), special education (12 years of special education)*. Blind children were assumed to live at home to avoid expensive residential care	n/a	No
Stillwaggon^20^	Cost of developmental disabilities taken from literature (Honeycutt paper)	Direct medical costs *(screening, blood testing, treatment*), *non-medical services and equipment* - not adequately described and indirect costs: Productivity losses	n/a	Yes: one-way and two-way (deterministic) sensitivity analysis
**Retinal dystrophy (RPE-65 mediated)**				
Viriato^60^	Attached national reference and unit costs, cost assigned to VC was list price, costs described in detail in supplementary material	Health costs associated with treatment procedure based on trial, including - *cost of VN, administration, monitoring, eligibility testing and adverse events*	Phase 3, open label RCT of voretigene neparvovec vs control	Yes: univariate and probabilistic (Monte Carlo simulations) and scenario analyses
Zimmerman^59^	Costs taken from US based literature	Direct medical: *VN treatment, physician visits, ophthalmic tests*. Indirect costs: *additional education due to visual impairment & Productivity losses*. Direct nonmedical: *caregiver, transportation, nursing home costs*	Phase 3, open label RCT of voretigene neparvovec vs control	Yes, univariate and probabilistic
**Retinopathy of Prematurity**				
Brown^46^	Charges from current procedural terminology (CPT) data (state of Pennsylvania) in line with CRYO-ROP trial	Cost of *laser photocoagulation therapy, cryotherapy initial consultation, intensive care unit bed stays*	CRYO-ROP	Yes: univariate, only varied discount rate
_Dave_^26^	Hospital costs acquired from hospital NICU site visit in 2010. Indirect costs: Sum of total years of lost productivity (lifetime productivity loss) - lost GDP per capita used to measure lost productivity of blind individual. - sex-adjusted. Opportunity cost of informal care given by family member who cares for blind individual used average hours of caregiving from survey of 35 parents with blind children in Lima.	Direct and Indirect costs included. Direct: *ROP screening, treatment, follow up clinic visits, medical and nursing staff costs*. Indirect costs*: lifetime productivity losses of the blind individual, productivity losses of caregiver*	CRYO-ROP & ETROP for long term outcomes	No
Dunbar^34^	Reimbursement rates from Medicaid providers - using procedure codes (CPT) in 2006	Medical costs: *pan retinal photocoagulation treatment, inpatient consultations, ‘retinal drawings’ (anaesthesiology not included)*	CRYO-ROP & ETROP	No
Jackson^47^	Medicare charges (from 2006 used) taken from laser photocoagulation, fundus photography, ophthalmoscopy.	Medical costs associated with management and treatment of ROP: cost of each ‘exam’ type; *fundus photography, ophthalmoscopy (inpatient consultation, hospital care, laser photocoagulation)*	CRYO-ROP & ETROP for long term outcomes	One way only
Javitt^42^	Costs used from Medicare charges from Healthcare Financing Administration (national), assumed is national program of screening was available - that would cost same as current Medicare reimbursement rates.	Direct medical costs of ROP screening and treatment (consultations, general anaesthesia, *ophthalmic follow up, physician costs for cryotherapy: per eye)* and costs of government delivered programs *(special education, training, social support payment etc*)	CRYO-ROP	Yes: univariate only
Kamholz^48^	Hospital costs taken from charges (converted using department specific cost to charge ratios)	Direct medical costs only: Hospital costs - *NICU admission, physician care (ophthalmologist)*	ETROP	Probabilistic and deterministic analysis
Naguib^39^	Costs of screening came from ‘in country’ hospital data. Productivity losses by caregiver calculated using national GDP per hour. Other costs collected from family surveys	Direct and indirect costs included. Direct costs: *screening, equipment, nursing & labour costs. ROP treatment: pan retinal photocoagulation and standard care, physician visits*. Indirect costs: *productivity losses of caregiver and blind individual*	CRYO-ROP & ETROP for long term outcomes	Yes: deterministic and probabilistic analysis (Monte Carlo simulation)
Rothschild^40^	Physician costs taken from published report from a medical association, nursing salaries from bureau of labour statistics. Productivity losses based on GDP per capita estimates	Direct medical costs: *screening, equipment, labour physician, nursing)* for screening, treatment & follow up care. Non-medical costs: *equipment, education*. Indirect costs: productivity losses of caregiver due to raising a blind child	CRYO-ROP & ETROP for long term outcomes	Yes: deterministic and probabilistic analysis (Monte Carlo simulation) and scenario analysis & threshold analysis
Zin^19^	Cost data on equipment from city’s ministry of health (staff salaries) and equipment costs from suppliers.	Medical costs of screening and treatment of ROP: *staff (ophthalmologist, anaesthesiologist, neonatologists, nurses), equipment for screening and treatment of ROP in NICU (indirect ophthalmoscope, neonatal depressor, indirect diode laser)* maintenance and calibration costs of equipment included. Also included cost of training ophthalmologists to detect and treat ROP.	n/a	yes: one and two way deterministic analysis of base case values (increased by 30% decreased by 36% - high and low range)
***ALL CAUSE VISION LOSS***				
Australia Save Sight Institute - University of Sydney^35^	Diagnosis-related group cost weights used to calculate hospital admission costs due to eye conditions. Productivity losses (from absenteesim & lower workforce participation) calculated using national average wage rate, employment rate and average weekly earnings.	Medical costs: hospital admitted services, out of hospital medical services, pharmaceuticals requiring a prescription and research for eye diseases. Non-medical costs: informal care costs, aids and modifications. Indirect costs included productivity losses for parents with children with VI due to unemployment and absenteeism, and deadweight losses (welfare payments).	n/a	No
Guide Dogs^17^	Costs based on expert opinion and from the literature	Non-treatment costs’: including disability living allowance, carer’s allowance, vision rehab services, income support, job seeker’s allowance, tax allowances; the provision of community based healthcare and the numbers of blind and partially sighted people unemployed	n/a	No
Honeycutt^15^	Hospital and medical (physician) costs from national medical expenditure survey, home and auto modifications from national health survey and vendor prices, Special education from disability surveillance program and the literature. Productivity losses due to disability from national survey of Income and Program Participation and the literature. Premature mortality was calculated using data from the literature (sex and age specific earnings and value of household productivity to calculated average annual productivity)	Direct medical costs (physician visits, medications, hospital inpatient stays, assistive devices, therapy, rehab, long term care/ non-medical costs (home and automobile modifications), special education, and Indirect costs: lost earnings of blind individuals due to disability and preterm mortality	n/a	Yes, univariate - varied discount rate only
Korvenranta^27^	National reference costs used from National Institute for Health and Welfare	Direct healthcare costs: inpatient hospital, outpatient (emergency & nonemergency), primary care, health professional visits (including therapists), social welfare services	n/a	No
O’Connor^30^	no info on unit costs used in paper only in main paper. All areas were reported as costs (expenditures) aside from informal care-which was collected in hours.	Personal costs’-4 main categories of resource use/cost collected: 1) medicines, products and equipment 2) health and community services 3) informal care & support and 4) other expenditure	n/a	No
Shamanna^41^	GNP per capita data for India. Cost of cataract treatment taken from the literature (cost of surgery in community ‘eye camp’) and guidelines from Word Bank Assistance Cataract Blindness Project	For children-measured ‘indirect costs’ to family associated with caring for the child who is blind (productivity losses of family member). Also included estimate cost of treating cataract associated blindness	n/a	No
Wittenborn^18^	Expenditure data taken from private insurance claims (Marketscan-used average outpatient cost for each ICD eye diagnosis), self-reported costs for optometry visits and vision aids (glasses & contact lenses). Informal care costs calculated using US average wage rate. Cost of SEN programmes, screening etc taken from US registries and the literature. Productivity losses based on difference in average income among those reporting different levels of visual functionality (Survey of Income and Program Participation). Other costs taken from the literature and federal budgets	Medical costs (attributable to eye disorders): private insurance claims relating to eye diagnoses (no further detail), cost of glasses, low vision aids and devices, caregiving, special education, (school entry) vision screening, federal assistance programs. Indirect costs: deadweight losses (transfer payments), productivity losses (productivity losses of child caregivers and adult with VI productivity losses) and loss of wellbeing	n/a	Yes: one way (of all major parameters) and probabilistic (simultaneously varied all major parameters in Monte Carlo simulation)
Wittenborn^38^	Expenditure data taken from private insurance claims (Marketscan-used average outpatient cost for each ICD eye diagnosis), self-reported costs for optometry visits and vision aids (glasses & contact lenses). Informal care costs calculated using US average wage rate. Cost of SEN programmes, screening etc taken from US registries and the literature. Productivity losses based on difference in average income among those reporting different levels of visual functionality (Survey of Income and Program Participation). Other costs taken from the literature and federal budgets	Direct costs: medical costs of diagnosed eye disorders, medical vision aids, low vision aids (eyeglasses & contacts), vision correction (optometry visits) special education, school entry vision screening, federal assistance programs. Indirect costs: productivity losses of caregivers, long-term care, transfer payments (not included in total), deadweight losses from transfer payments	n/a	Yes: one way (varied high and low) and probabilistic (Monte Carlo simulation) - all variables varied
Wright^31^	No description	“Direct financial costs”: pension, carer allowance, subsidies (pharmaceutical supplement, household, travel concessions, rent concessions, equipment (education and employment related) and low vision services (including low vision clinic, mobility services, occupational therapy, general rehab, day centre use, visiting teacher services)	n/a	Says sensitivity analysis was undertaken but only to present estimated ‘lower and upper’ cost. Very limited information on SA

CRYOROP: Multi-centre Trial of Cryo-therapy for Retinopathy of Prematurity ETROP: Early treatment of Retinopathy of Prematurity Trial NICU: Neonatal Intensive Care Unit GDP: Gross Domestic Product GNP: Gross National Product SEN: Special Educational Needs ICD: International Classification of Diseases SA: Sensitivity analysis SYCAMORE: Safety and Cost-effectiveness of Adalimumab in combination with Methotrexate for the treatment of juvenile idiopathic arthritis

**Table 3:** Disaggregated total annual costs (in 2020 USD) *per child

First author	Ophthalmic condition	Analytic perspective	Time horizon	Description of main cost measure	Main cost estimate (2020 USD)	Direct medical (pp)	Direct nonmedical (pp)	Indirect cost (pp)
**WHOLE GLOBE AND ANTERIOR SEGMENT**								
**CHARGE syndrome**								
Anderzen-Carlsson^29^	CHARGE syndrome	Healthcare system	1 year (1st year of life)	N/A	N/A	N/A		
**Glaucoma**								
_Liu_^37^	Primary childhood glaucoma (PCG) and secondary glaucoma(SCG)	Payer	4 years since diagnosis	Total cost of illness of glaucoma cohort (until 4 years since dx)	$100,534			
**LENS** (Cataract)								
Cernat^22^	Cataract (Bilateral)	Healthcare system	8 weeks	Cost saving per patient	$5,029	$2,930		
Dave^24^	Cataract (congenital, bilateral)	Societal	Till 4 years old	Societal lifetime cost	$82	$2,950		$233,695
Wang^28^	Cataract - Bilateral developmental non-traumatic paediatric	Patient	n/a - cost of surgery only	Mean cost during treatment*	$4,491			
**UVEA** (Uveitis)								
Hughes^49^	JIA Uveitis	Healthcare system	18 months	Incremental cost/QALY	$96,668	$101,299		
Minden^25^	JIA Uveitis	Societal	1 year (12 months)	Mean cost of JIA with ‘eye involvement’ (€5,146 (SD: 6.969), median IQR: €2,407 (865-6254)	$4,809			
Noble^21^	Nongranulomatous anterior uveitis	Healthcare system	? One year	Cost savings per child	$18	$18		
***RETINA***								
**Congenital toxoplasmosis**								
Prusa^23^	congenital toxoplasmosis (retinochoroiditis, blindness)	Societal	Lifetime (max age not defined in main paper)	Cost saving per birth	$299			
Roberts & Frenkel^36^	congenital toxoplasmosis	Societal	Lifetime	Total cost of illness (till 18 yrs) per child	$2,105,640	$91,524	$641,489	$13,708,341
Stillwaggon	congenital toxoplasmosis	Societal	Lifetime (76 yrs)	Cost savings per child	$747			
**Retinal dystrophy (RPE-65 mediated)**								
Viriato^60^	Inherited retinal dystrophy (biallelic, RPE-65 mediated)	Healthcare system	Lifetime (treatment effect of approx. 70 years)	ICER (Cost per QALY)	$70,452			
Zimmerman^59^	Inherited retinal disease (biallelic RPE-65 mediated)	Societal	Lifetime (treatment effect of approx 70 years)	ICER (Cost per QALY)	$531,018			
**Retinopathy of Prematurity (ROP)**								
Brown^46^	ROP - Threshold (proliferative retinal vascular disease that is located in zone 1 or 2 in conjunction with plus disease and associated with 5 contiguous or 8 cumulative clock hours of extraretinal neovascularization)	Payer	Lifetime (76 years)	Cost/QALY	$2,678			
Dave^26^	ROP associated blindness (bilateral) - uses WHO definition of blindness & low vision (<20/60 BCVA in better eye). From trial data used must be threshold ROP	Payer	Lifetime: 20 years	Cost difference between treatments (per child)	$2,790			
Dunbar^34^	ROP (threshold)	Payer	Lifetime (77.5)	Cost/QALY	$1,881			
Jackson^47^	ROP (Threshold): unilateral and bilateral	Payer	Lifetime (77.5)	Cost/QALY (lifetime cost)	$4,024			
Javitt^42^	ROP (threshold)	Other	1 year (1st year of life)	Cost/QALY	$4,103			
Kamholz^48^	ROP (high risk prethreshold ROP)	Payer	9 months old (adjusted gestational age), also estimated lifetime costs (77.6 yrs)	ICER per eye with SVI prevented	$18,448			
Naguib^39^	ROP related blindness	Societal	Lifetime	Lifetime cost of illness	$107,186			$85,651
Rothschild^40^	ROP	Societal	Lifetime	Incremental net monetary benefit (INMB)	$6,100			
Zin^19^	ROP (birthweight <1.5kg)	Healthcare system	1 year	Incremental cost/infant treated	$94	$94		
**ALL CAUSE VISUAL IMPAIRMENT**								
Australia Save Sight Insitutute - University of Sydney^3^	All childhood eye conditions that result in ‘blindness or severe vision impairment’ - including unilateral (amblyopia) as well as correctable refractive error	Societal	Lifetime	Lifetime health system expenditure per child	$45,189	$2,009		$26,308,517
Guide Dogs^17^	“congenital visual impairment, e.g. Retinitis pigmentosa”, **otherwise undefined**	Societal	NA	Lifetime cost	$260,595			$158,963
Honeycutt^15^	Vision impairment – **undefined**	Societal	Lifetime	Total cost of illness pp (x- sectional estimate)	$682,412	$46,633	$69,278	$566,500
Korvenranta	Blindness & visual disturbances in very premature infants	Healthcare system	1 year: costs at 5^th^ year of life	Eye specific cost: Marginal cost of visual disturbance or blindness	$1,177	$724		
O’Connor^30^	Bilateral moderate visual impairment (VI),bilateral blindness (BL),mild vision loss (6/7.5, 6/9)	Patient	1 year	N/A	N/A	N/A		
Shamanna^41^	Childhood blindness **- undefined**	Not reported	None - crosssectional	Estimated GNP lost (of population) (in billions)	$30,041			$30,030
Wittenborn^18^	VI (all cause mild (>20/40), moderate vision impairment (>20/80) and blindness(20/200))	Societal	none - crosssectional	Total cost (of population)	$6,723,640,929			$687,128,845
Wittenborn^38^	Includes 1) costs of diagnosed eye disorders, 2) undiagnosed selfreported vision loss and 3) vision correction.	Societal	none - crosssectional	Total cost (of population)	$6,512,391,400			$723,275,857
Wright^31^	“Congenital visual impairment” **- undefined**	Payer	1 year (annual costs)	Total cost of illness (till first 18 yrs)	$41,637	$119,942		$1,796,477

ICER: incremental cost effectiveness

## Key points

- Childhood visual impairment and blindness (VI/SVIBL) results in permanent visual disability in both eyes and affects all aspects of development. As such it is has a significant socio-economic impact on the child, family and society. Health economic data on disorders resulting in childhood VI/SVIBL are necessary for effective and equitable planning and provision of specialist health care and support.
- This is the first systematic literature review to evaluate the quantity and quality of studies reporting resource use and costs associated with disorders that result in childhood visual disability. We found that the health economic literature on childhood VI/SVIBL disorders is sparse. Studies in retinopathy of prematurity (ROP) were most frequently studies in the literature, whereas no studies were carried out in cerebral visual impairment (CVI) and optic nerve disorders.
- We highlight a significant evidence gap in the health economic literature of childhood VI/SVIBL. It is crucial that visual outcomes are included as core outcome measures of future trials so these data are available for cost-effectiveness models. Future economic evaluations and cost-of-illness studies of blinding childhood disorders can be improved by enhanced reporting of key data items and definitions.
