# Supplementary material for: Evaluating the quantity and quality of health economic literature in blinding childhood disorders: a systematic literature review
Source: Pharmacoeconomics. Author manuscript; Available in PMC 2024 Mar 1. (PMC7615631; doi:10.1007/s40273-023-01311-5)
Supplement: Supplementary information [file EMS191282-supplement-Supplementary_information.docx]

**Supplementary information**

**Figure S1. Search strategy for PUBMED**

**(child[MeSH Terms]) OR ((paediatric[Title/Abstract]) OR pediatric[Title/Abstract])) OR ((((((((((Child*[Title/Abstract]) OR infant*[Title/Abstract]) OR Newborn*[Title/Abstract]) OR Baby[Title/Abstract]) OR Babies[Title/Abstract]) OR Neonat*[Title/Abstract]) OR Preterm*[Title/Abstract]) OR Prematur*[Title/Abstract]) OR Schoolchild*[Title/Abstract]) OR Preschool[Title/Abstract] OR Toddler*[Title/Abstract] OR Adolesc*[Title/Abstract] OR Teen*[Title/Abstract] OR Pubert*[Title/Abstract] OR Kindergar*[Title/Abstract])**

**AND**

**(eye diseases[MeSH Terms]) OR vision disorders[MeSH Terms]) OR blindness[MeSH Terms]) OR visually impaired persons[MeSH Terms]))))) OR "blindness"[Title/Abstract]) OR "vision loss"[Title/Abstract]) OR "vision impair*"[Title/Abstract]) OR "visual impair*"[Title/Abstract]))))))**

**AND**

**(cost of illness[MeSH]) OR ("Health Care Costs/statistics and numerical data"[MeSH])) OR (health care costs[MeSH]) OR "economic cost"[Title/Abstract]) OR "financial burden"[Title/Abstract]) OR (Blindness/economics[MeSH Major Topic])) OR (low vision/ economics[MeSH Major Topic]))))**

**NOT**

**(Review[Publication Type]))) NOT (Meta-Analysis[Publication Type]))**

**Figure S2. Grey literature search strategy**

Search terms used in Google search engine:

1. Economic cost of childhood visual impairment UK
2. Economic cost of childhood visual impairment
3. Economic cost of child visual impairment
4. Economic impact and cost visual impairment child
5. Economic impact and cost of childhood blindness

Citations from first 10 pages of results were compiled in Mendeley then exported into Covidence for removal of duplicates and screening.

**Table S1 items collected in data extraction form**

1. First author
2. Year of publication
3. Country
4. Time/study period
5. Eye disorder or VI level
6. Paediatric age range (years)
7. Sample size (number of children <19 years old)
8. Analytic perspective
9. Costing approach
10. Study design
11. Time horizon
12. Name of randomised controlled trial if used trial data
13. Cost estimates
14. Data source(s)
15. Resource valuation
16. Cost components
17. Currency, year
18. Sensitivity analysis
19. Was health related quality of life (HRQoL) measured?

**Table S2 Quality assessment scoring scheme for cost of illness studies**

| Item | Question |
| --- | --- |
| **1** | Was a clear definition of the illness given? |
| **2** | Were epidemiological sources carefully described? |
| **3** | Were (direct/indirect) costs sufficiently disaggregated ? |
| **4** | Were resource use data sources carefully described? |
| **5** | Were resource use data appropriately assessed? |
| **6** | Were the sources of all cost values analytically described? |
| **7** | Were unit costs appropriately valued? |
| **8** | Were the methods adopted carefully explained? |
| **9** | Were costs discounted ? |
| **10** | Were the major assumptions tested in a sensitivity analysis? |
| **11** | Was the presentation of study results consistent with the methodology of study? |

Each question in the checklist was of equal weight, and scored as Yes, No or Partially or not applicable. The total score was the sum of across all 11 items, Yes was 1 point, No was 0 points and Partially was 0.5. Articles with items that were ‘not applicable’ were removed from the denominator in calculating the total score.

**Table S3a. Quality assessment scores for studies for case studies**

|  | **Molinier checklist for cost of illness studies** | **O’Connor** | **Wright** | **Anderzen-Carlsson** |
| --- | --- | --- | --- | --- |
| Item | Question | Case studies | | |
| **1** | Was a clear definition of the illness given? | Yes | No | Yes |
| **2** | Were epidemiological sources carefully described? | P | No | Yes |
| **3** | Were (direct/indirect) costs sufficiently disaggregated ? | P |  |  |
| **4** | Were resource use data sources carefully described? | P | P | P |
| **5** | Were resource use data appropriately assessed? | No | P | P |
| **6** | Were the sources of all cost values analytically described? | No | Yes |  |
| **7** | Were unit costs appropriately valued? | No | P |  |
| **8** | Were the methods adopted carefully explained? | P | P | Yes |
| **9** | Were costs discounted ? |  | No |  |
| **10** | Were the major assumptions tested in a sensitivity analysis? |  | P | No |
| **11** | Was the presentation of study results consistent with the methodology of study? | P | Yes | Yes |
|  | **Total score by study** |  |  |  |
|  | Total Yes | 1 | 2 | 4 |
|  | Total Partial | 4 | 5 | 2 |
|  | Total No | 3 | 3 | 1 |
|  | blank is NA | 2 | 1 | 4 |
|  |  | 1.5 | 3.5 | 1 |
|  |  | 39% | 45% | 71% |

Legend

N/A= not applicable

**Table S3b. Quality assessment scores for studies for economics evaluations (EEs)**

| **Item** | Stillwa  ggon | Viriato | Hughes | Zimme  rman | Cernat | Naguib | Roth  schild | Jack  son | Dunbar | Kamholz | Zin | Brown | Javitt | Dave | Prusa | Noble |
| --- | --- | --- | --- | --- | --- | --- | --- | --- | --- | --- | --- | --- | --- | --- | --- | --- |
|  | Economic Evaluation | | | | | | | | | | | | | | | |
| **1** | Yes | Yes | Yes | Yes | P | P | P | P | P | P | P | Yes | P | Yes | Yes | Yes |
| **2** | P | P | Yes | Yes | P | P | Yes | Yes | Yes | P | Yes | Yes | Yes | Yes | Yes | Yes |
| **3** | No |  |  | Yes | Yes | Yes | No |  |  |  |  |  |  | Yes | P |  |
| **4** | P | Yes | Yes | Yes | Yes | P | P | P | Yes | Yes | Yes | Yes | Yes | Yes | Yes | P |
| **5** | Yes | Yes | Yes | P | Yes | P | Yes | P | Yes | Yes | Yes | Yes | P | Yes | Yes | Yes |
| **6** | Yes | Yes | Yes | Yes | Yes | P | P | Yes | Yes | Yes | P | Yes | Yes | Yes | Yes | P |
| **7** | P | Yes | Yes | Yes | Yes | Yes | P | Yes | Yes | Yes | Yes | Yes | Yes | Yes | Yes | Yes |
| **8** | Yes | Yes | Yes | Yes | Yes | P | P | Yes | Yes | Yes | Yes | Yes | Yes | Yes | Yes | Yes |
| **9** | Yes | Yes | Yes | Yes | No | No | Yes | No | No | No | Yes | No | Yes | No | Yes | No |
| **10** | Yes | Yes | Yes | Yes | Yes | No | Yes | Yes | No | Yes | Yes | Yes | Yes | No | Yes | No |
| **11** | Yes | Yes | Yes | Yes | Yes | Yes | Yes | Yes | Yes | Yes | Yes | Yes | Yes | Yes | Yes | Yes |
| Scores |  |  |  |  |  |  |  |  |  |  |  |  |  |  |  |  |
| Yes | 7 | 9 | 10 | 10 | 8 | 3 | 5 | 6 | 7 | 7 | 8 | 9 | 8 | 9 | 10 | 6 |
| Partial | 3 | 1 | 0 | 1 | 2 | 6 | 5 | 3 | 1 | 2 | 2 | 0 | 2 | 0 | 1 | 2 |
| No | 1 | 0 | 0 | 0 | 1 | 2 | 1 | 1 | 2 | 1 | 0 | 1 | 0 | 2 | 0 | 2 |
| N/A | 0 | 1 | 1 | 0 | 0 | 0 | 0 | 1 | 1 | 1 | 1 | 1 | 1 | 0 | 0 | 1 |
| Total items | 8.5 | 8.5 | 9.0 | 10.5 | 9.0 | 6.0 | 7.5 | 6.5 | 6.5 | 7.0 | 8.0 | 8.0 | 8.0 | 9.0 | 10.5 | 6.0 |
| Total score* (%) | 77% | 77% | 77% | 77% | 77% | 77% | 77% | 77% | 77% | 77% | 77% | 77% | 77% | 77% | 77% | 77% |

*Total score as a percentage (Total score of items/Total number of answered items)

Legend

N/A= not applicable

**Table S3c. Quality assessment scores for studies for cost of illness (COI) studies**

|  | **Dave** | **Shamanna** | **Roberts** | **Wang** | **Liu** | **Wittenborn** | **Korvenranta** | **Minden** |
| --- | --- | --- | --- | --- | --- | --- | --- | --- |
| Item | Cost of Illness studies | | | | | | | |
| **1** | P | Yes | No | Yes | Yes | Yes | Yes | No |
| **2** | Yes | P | Yes | P | Yes | P | Yes | Yes |
| **3** |  |  | Yes | Yes | Yes | Yes |  | P |
| **4** | Yes | P | P | Yes | P | P | Yes | Yes |
| **5** | Yes | Yes | Yes | Yes | Yes | Yes | Yes |  |
| **6** | Yes | P | P | Yes | P | Yes | Yes | P |
| **7** | Yes | Yes | Yes | Yes | Yes | Yes | Yes | Yes |
| **8** | Yes | Yes | Yes | P | Yes | Yes | Yes | Yes |
| **9** | No | No | Yes | No | Yes | No | Yes | Yes |
| **10** | No | No | No | No | No | Yes | No | Yes |
| **11** | Yes | Yes | Yes | Yes | Yes | Yes | Yes | Yes |
| Scores |  |  |  |  |  |  |  |  |
| Yes | 7 | 5 | 7 | 7 | 8 | 8 | 9 | 7 |
| Partial | 1 | 3 | 7 | 2 | 2 | 2 | 0 | 2 |
| No | 2 | 2 | 2 | 2 | 1 | 1 | 1 | 1 |
| N/A | 1 | 1 | 0 | 0 | 0 | 0 | 1 | 1 |
| **Total item score** | **6.5** | **5.5** | **8** | **8** | **9** | **9** | **8** | **7** |
| **Total % score* (%)** | **75%** | **65%** | **73%** | **73%** | **82%** | **82%** | **90%** | **80%** |

*Total % score as a percentage (Total score of items/Total number of answered items)

Legend

N/A= not applicable

**Table S3d. Quality assessment scores for studies for studies from the grey literature**

|  | **Guide Dogs** | **Honeycutt** | **Australia Save Sight Institute - University of Sydney** | **Wittenborn** |
| --- | --- | --- | --- | --- |
| Item | Grey literature | | | |
| **1** | No | No | P | Yes |
| **2** | No | Yes | Yes | P |
| **3** |  | Yes | Yes | Yes |
| **4** | No | Yes | P | P |
| **5** |  | Yes | P | P |
| **6** | Yes | Yes | P | P |
| **7** | P | P | Yes | Yes |
| **8** | No | Yes | P | Yes |
| **9** |  | Yes | Yes | No |
| **10** | No | P | No | Yes |
| **11** |  | Yes | P | Yes |
| Scores |  |  |  |  |
| Yes | 1 | 8 | 4 | 6 |
| Partial | 1 | 2 | 5 | 4 |
| No | 5 | 1 | 1 | 1 |
| N/A | 4 | 0 | 0 | 0 |
| **Total item score** | **-2.5** | **9** | **7** | **8** |
| **Total % score* (%)** | **21%** | **82%** | **64%** | **73%** |

*Total % score as a percentage (Total score of items/Total number of answered items)

Legend

N/A= not applicable
